# Boron Nitride Nanosheets/PNIPAM Hydrogels with Improved Thermo-Responsive Performance

**DOI:** 10.3390/ma11071069

**Published:** 2018-06-24

**Authors:** Shishan Xue, Yuanpeng Wu, Jiemin Wang, Meiling Guo, Dan Liu, Weiwei Lei

**Affiliations:** 1 School of Material Science and Engineering, Southwest Petroleum University, Chengdu 610500, China; 201711000008@stu.swpu.edu.cn (S.X.); 201621000011@stu.swpu.edu.cn (M.G.); 2 State Key Laboratory of Oil and Gas Reservoir Geology and Exploitation, Southwest Petroleum University, Chengdu 610500, China; 3 Institute for Frontier Materials, Deakin University, Locked Bag 2000, Geelong, VIC 3220, Australia; jiemin@deakin.edu.au

**Keywords:** thermal-responsive, hydrogel, boron nitride nanosheets, poly(*N-*isopropylacrylamide)

## Abstract

Thermo-responsive hydrogel is an important smart material. However, its slow thermal response rate limits the scope of its applications. Boron nitride nanosheet-reinforced thermos-responsive hydrogels, which can be controlled by heating, were fabricated by in situ polymerization of *N-*isopropylacrylamide in the presence of boron nitride nanosheets. The hydrogels exhibit excellent thermo-responsiveness and much enhanced thermal response rate than that of pure poly(*N-*isopropylacrylamide) hydrogels. Interestingly, the hydrogels can be driven to move in aqueous solution by heating. Importantly, the composite hydrogel is hydrophilic at a temperature below lower critical solution temperature (LCST), while it is hydrophobic at a temperature above LCST. Therefore, it can be used for quick absorption and release of dyes and oils from water. All these properties demonstrate the potential of hydrogel composites for water purification and treatment.

## 1. Introduction

Heat transport is important in biomedical and biotechnological processes [1]. Extreme heating such as ablation can be used to destroy cancers and tumors, while cooling of organs destined for transplantation prevents cell and tissue damage [2]. So heat conduction rate plays a key role in the process of treatment of bad tissues [3]. Hydrogel is a three-dimensional cross-linked polymeric network [4] and can swell in aqueous medium [5]. Smart hydrogels, also called “the stimulus responsive hydrogels”, have attracted much academic and industrial attention due to their excellent external stimuli responsive properties, such as temperature, pH, photons, or magnetic responsiveness [6].

Among them, the environmentally thermo-responsive poly(*N-*isopropylacrylamide) (PNIPAM) hydrogel has the most potential since it is reversible from hydrophilicity to hydrophobicity when the temperature reaches the lower critical solution temperature (LCST) of around 32 °C [7,8,9,10]. Such smart properties endow it with diverse applications in biomedicine [11,12], drug delivery [13,14], hydrogen storage [15,16] and field emitting [17,18]. However, the slow thermal responsive rate upon heating/cooling across the LCST of PNIPAM severely limits their applications. To improve the response rate of PNIPAM-based thermo-responsive hydrogel, different strategies have been developed, such as modifying the structures of hydrogels by incorporating nano-fillers [10,11,12]. Recent work reveals that the combination of two-dimensional (2D) nanomaterials, such as grapheme and MoS_2_, with PNIPAM hydrogels results in an impressive thermo-response rate [19,20,21,22]. Therefore, searching for new 2D candidate compatibility with PNIPAM is still an urgent task.

Boron nitride nanosheet (BNNS), also called “white graphene” [23], possesses excellent thermal conductivity (~6000 W·m^−1^·K^−1^) [24], significant chemical inertness [25], uniquely mechanical properties [26], high resistant to oxidation (stable up to 840 °C in air), low dielectric constant [27], and exceptional electrical insulativity [28]. Such outstanding properties make it highly promising in a wide range of applications such as field nano-emitters, hydrogen storage, organic pollutant adsorption and clean-up of oil spillage [14]. However, to the best of our knowledge, only a few investigations have been devoted to preparing smart nanocomposites hydrogels with BNNS for improving the thermal response. Most recently, Xiao [25] reported that exfoliated hydroxyl functionalized-BNNS (BNNS-OH) which have been incorporated into PNIPAM hydrogels for enhancing thermal response. However, the low concentration (0.06 mg/mL) of BNNS-OH in ethanol by a steam treatment method [25] may present a severe limitation for the aqueous suspensions preferred in many practical applications.

**Scheme 1:**
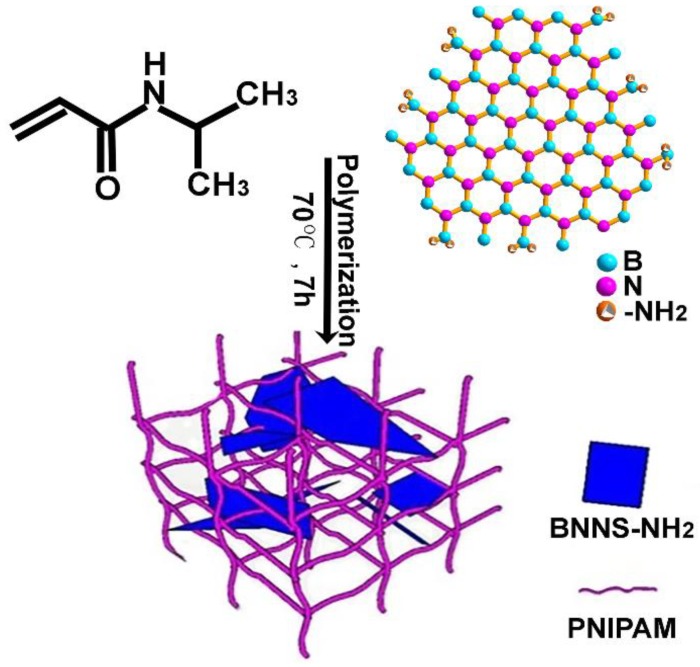
Schematic illustration of the preparation of PNIPAM/BNNS-NH_2_ hydrogels.

In this work, the as-produced few-layer amino functionalized-BNNS (BNNS-NH_2_) with high dispersibility in water, yielding stable colloidal solutions with concentrations up to 0.20 mg/mL are introduced into the thermally responsive hydrogels, PNIPAM (Scheme 1) [14]. The composite hydrogels exhibit more sensitive property to temperature change. Moreover, the smart composite hydrogel shows extraordinary large responsive swelling ratio and rapid response rate. Importantly, the resulting smart hydrogel demonstrates the potential of oil and dye desorption upon heating process.

## 2. Materials and Methods

### 2.1. Materials

The *N-*isopropylacrylamide (NIPAM), amino functionalized boron nitride nanosheet (BNNS-NH_2_) are used as reactants, the *N*,*N*′-methylenebisacrylamide (BIS) and the azobisisobutyronitrile (AIBN) are used as crosslinking agent and initiator respectively. The NIPAM, BIS and AIBN were purchased from Sigma-Aldrich (Saint Louis, MO, USA). The BNNS-NH_2_ was prepared by our previous work [14,29]. 

### 2.2. Synthesis of the PNIPAM Hydrogels Incorporated with BNNS-NH_2_

Different amounts of BNNS-NH_2_ were dispersed into alcohol and sonicated for 3 h and alcohol solution with the concentration of BNNS-NH_2_ equal to 0.02 mg/mL, 0.04 mg/mL, 0.06 mg/mL, 0.10 mg/mL and 0.20 mg/mL were obtained respectively. Then, NIPAM (0.6 g), BIS (20 mg) and AIBN (22 mg) were added into alcohol solution of BNNS-NH_2_ (3 mL) under stirring. The mixture was bubbled by N_2_ for 30 min and polymerized at 70 °C for 7 h to prepare PNIPAM/BNNS-NH_2_ hydrogels, as displayed in Scheme 1. The as-prepared hydrogels were then washed with alcohol and water 3 times. Pure PNIPAM hydrogels were prepared just as the similar route but without adding the BNNS-NH_2_.

## 3. Results and Discussion

The PNIPAM/BNNS-NH_2_ hydrogel was prepared with the concentration of BNNS-NH_2_ tuned from 0.02 to 0.20 mg/mL. The hydrogels were characterized by Fourier transform infrared spectroscopy (FT-IR). As shown in Figure S1a,c, the weak but obvious band around 1930.3 cm^−1^ corresponds to the out of plane bending vibration of B-N-B, and the characteristic peaks of in-plane B-N stretching vibrations at 1314.8 cm^−1^ [14]. Figure S1b is FT-IR of pure PNIPAM hydrogel, the adsorption at 1556.3 cm^−1^ and 1625.7 cm^−1^ are assigned to stretching vibration of characteristic amide group and carbonyl moiety in [–C(O)–NH–]. The band at 2978.6 cm^−1^ is due to the asymmetric vibration of C–H in –CH_3_. The FT-IR of PNIPAM/BNNS-NH_2_ hydrogels was shown in Figure S1c, the characteristic peaks at 1314.8 cm^−1^ is correlated with in-plane B-N stretching vibrations. The band at the range of 861.5–950.3 cm^−1^ is correlated with out-of-plane bending mode of h-BN [25], which illustrates that the h-BN is not thoroughly exfoliated to the BNNS, or some BNNSs re-agglomerate in the polymer array to be the bulk BN.

To determine the mass fraction of polymer in PNIPAM/BNNS-NH_2_ hydrogel, thermos-gravimetric analysis (TGA) is tested. As shown in Figure S2, the BNNS-NH_2_ is stable in air up to 800 °C, any weight loss below that temperature is caused by the decomposition of amino groups [14]. The results of TGA illustrates that the composite hydrogel is chemical stably below 300 °C which is important in practical applications. In addition, for PNIPAM/BNNS-NH_2_ hydrogel prepared from BNNS-NH_2_ solution with concentration of 0.06 mg/mL, the weight loss of 7.7% is due to water in the hydrogel, and the residual weight loss of 83.7% is because of thermal degradation of PNIPAM.

**Figure 1:**
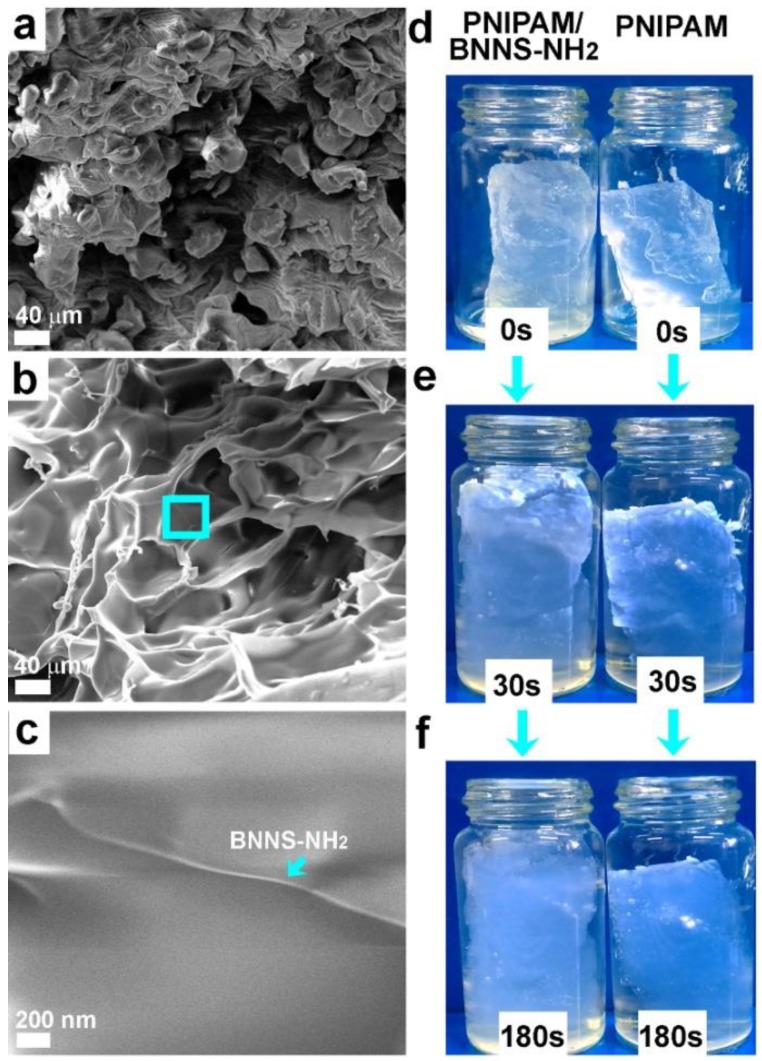
SEM images of the lyophilized hydrogels (**a**) PNIPAM, (**b**) PNIPAM/BNNS-NH_2_, and (**c**) magnification at cross-section in (**b**). Photographs of the hydrogels placed in hot water (42 °C) (the left one is PNIPAM/BNNS-NH_2_, the right one is pure PNIPAM): (**d**) before poured into hot water, (**e**) 30 s later and (**f**) 180 s later placed in hot water (42 °C).

The hydrogels with and without BNNS-NH_2_ were lyophilized and observed by scanning electron microscopy (SEM). As shown in Figure 1a–c, the pure PNIPAM exhibits the typical porous morphology, while the PNIPAM/BNNS-NH_2_ demonstrates the distinct porous structure with lamellar. The SEM images also show that BNNS-NH_2_ is clearly inside the porous PNIPAM/BNNS-NH_2_ hydrogel (Figure 1c). LCST of PNIPAM/BNNS-NH_2_ hydrogel is around 34 °C, as tested in Figure S3. Thermo-responsiveness of pure PNIPAM and PNIPAM/BNNS-NH_2_ hydrogels can be observed by poured the samples into hot water (42 °C) at the same time. As shown in Figure 1d–f, the PNIPAM/BNNS-NH_2_ hydrogel changes from transparent to turbid more quickly than that of PNIPAM hydrogel owing to respond to elevating of temperature more quickly in the presence of BNNS-NH_2_. This demonstrates that the heat transfer rate of PNIPAM/BNNS-NH_2_ hydrogel is effectively enhanced by BNNS-NH_2_. The composite hydrogel is more sensitive to the changing of temperatures, which is attributed to the superior thermal conductivity improved by introducing BNNS-NH_2_ [18].

**Figure 2:**
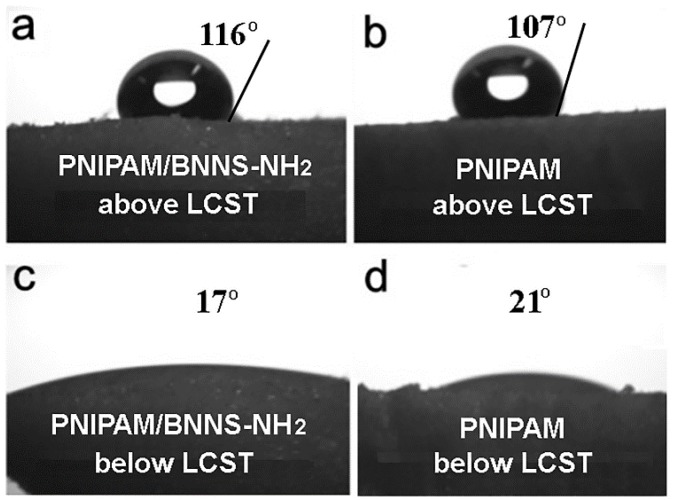
The pictures of contact angle: (**a**) PNIPAM/BNNS-NH_2_; (**b**) PNIPAM at above LCST; (**c**) PNIPAM/BNNS-NH_2_ and (**d**) PNIPAM at below LCST.

The hydrophilicity/hydrophobicity of the hydrogel surfaces plays an important role in the oil and dye adsorption. The water contact angles of the material surfaces were used to evaluate the hydrophilicity/hydrophobicity of hydrogel in Figure 2. It is clear that the contact angles of PNIPAM/BNNS-NH_2_ hydrogels is 116°at temperature above LCST (Figure 2a), which is more hydrophobic than pure PNIPAM (107°) as shown in Figure 2b. With the temperature decreasing to below LCST, the contact angle of the PNIPAM/BNNS-NH_2_ composite hydrogel decreases to 17° (Figure 2c), which is more hydrophilic than pure PNIPAM (21°) as shown in Figure 2d.

**Figure 3:**
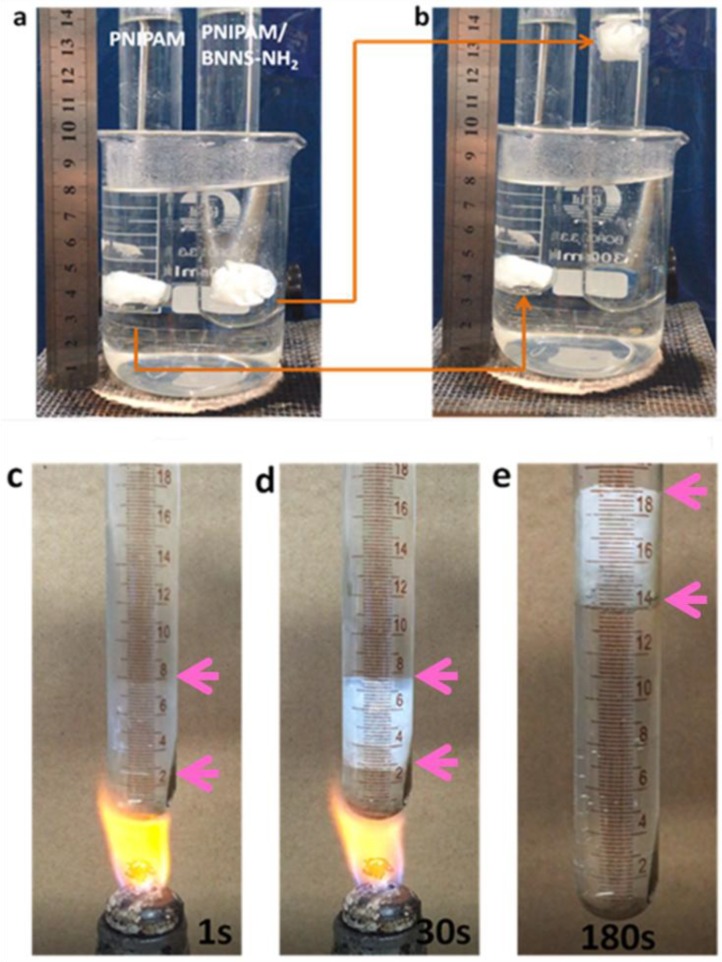
Digital photographs of hydrogels under heating: (**a**) at the beginning of heating, (**b**) heating for 10 min by a spirit lamp, PNIPAM/BNNS-NH_2_ hydrogels were heated for (**c**) 1 s, (**d**) 30 s and (**e**) 180 s from room temperature.

To demonstrate that the PNIPAM/BNNS-NH_2_ hydrogel possesses higher heat transfer efficiency than that of pristine PNIPAM, both the hydrogels were heated in the water bath at the same time, as shown in Figure 3. The PNIPAM/BNNS-NH_2_ hydrogel moves faster than that of pure PNIPAM hydrogel under heating (Figure 3a,b), indicating that BNNS-NH_2_ in the hydrogels enhances the thermal conductivity rate of the composite hydrogels. The PNIPAM/BNNS-NH_2_ hydrogel moves from bottom to top of cuvette under heating from room temperature to 40 °C. The polymer chains are hydrophilic and extensive at room temperature, making hydrogel stay at the bottom of cuvette (Figure 3c). Conversely, the polymer chains are hydrophobic and shrinking when the aqueous solution is heated to 40 °C (above LCST) (Figure 3d,e). During the heating, polymeric chains at the bottom of hydrogel are shrinking (high temperature) and the polymeric chains on the top are extension (low temperature), which impels the hydrogel to move from higher temperature to lower temperature (from bottom to top). Herein, the extension and shrinking of polymeric chains in the hydrogels [7] cause movement of hydrogels under heating in water. The PNIPAM/BNNS-NH_2_ hydrogels exhibit potential applications as actuators or intelligent delivery carriers in the fields of petroleum, information technology, environmental and medical science.

**Figure 4:**
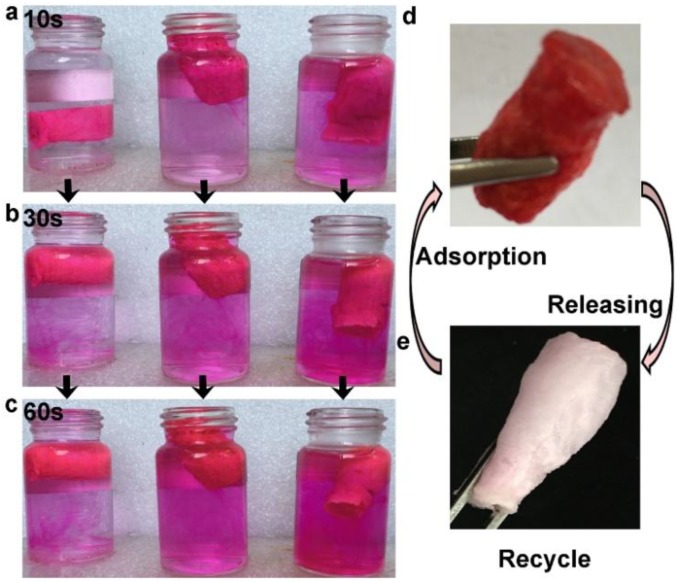
Photographs of dye release test, all samples were put in hot water for (**a**) 10 s, (**b**) 30 s and (**c**) 60 s. The bottles from left to right are pure PNIPAM and PNIPAM/BNNS-NH_2_ prepared from BNNS-NH_2_ concentration of 0.02 mg/mL and 0.04 mg/mL, respectively. Photographs of the PNIPAM/BNNS-NH_2_ hydrogel: (**d**) before and (**e**) after releasing dye.

The hydrogel can be employed to absorb and release hydrophilic molecules under the control of temperature. As revealed in Figure S4, the hydrogels absorb hydrophilic dye (Rhodamine B) in aqueous solution at room temperature (below LCST). PNIPAM/BNNS-NH_2_ hydrogels absorbed Rhodamine B faster than that of PNIPAM hydrogels, which probably because of BNNS-NH_2_ enhanced absorbing performance for dyes. In addition, the hydrogels which possess more BNNS-NH_2_ absorb dyes much faster, illustrating that BNNS-NH_2_ improves spaces and enhances H-bonds for absorbing more Rhodamine B [25]. The controlled releases of the absorbed Rhodamine B at high temperature (above LCST) are described in Figure 4 and Figure S5. The absorbed dyes can be released at high temperature and PNIPAM/BNNS-NH_2_ hydrogels release dyes faster than pure PNIPAM hydrogels. PNIPAM/BNNS-NH_2_ hydrogels containing more BNNS-NH_2_ exhibit faster releasing rate of absorbed dyes (Figure 4a–c). This may be attributed to the hydrogels with BNNS-NH_2_ which exhibit high thermal conductivity rate are more sensitive to heat. Importantly, the dyes in the composite hydrogels can be released completely after the hydrogels are put into the hot water for 10 h (Figure 4d,e), then the hydrogels can be recycled and reused many times, supporting a smart and recyclable equipment driven by temperature.

**Figure 5:**
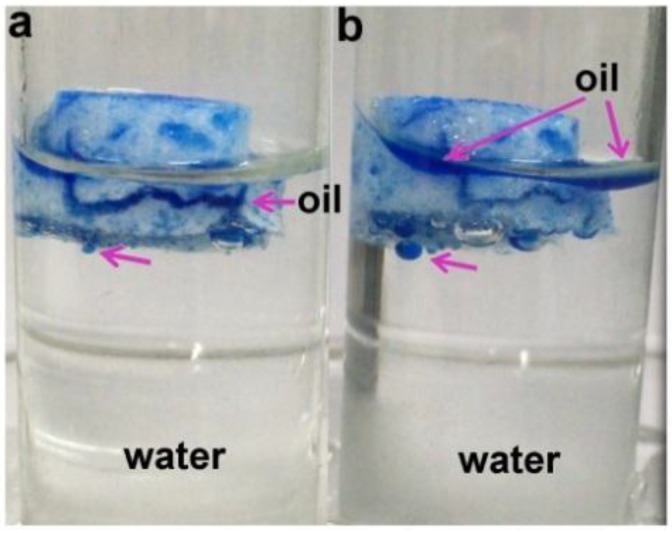
Photos of PNIPAM/BNNS-NH_2_ hydrogels were put into the cold water for 5 min (**a**) and for 10 min (**b**).

Significantly, the PNIPAM/BNNS-NH_2_ hydrogels with enhanced thermo-responsive property can also be used to absorb and release oleophilic molecules such as diesel by simply adjusting the temperatures. The absorption capacity for diesel is 3.7 g/g at temperature above LCST, comparable to most polymeric oil-absorbents [30,31]. As shown in Figure 5 the absorbed diesel (dyed by blue colorant) can be effectively released when the temperature was lower than LCST. The hydrophilic-lipophilic properties of the fabricated hydrogels at above and below LCST lead to the design of smart sorbents from the thermo-responsive hydrogels.

## 4. Conclusions

In conclusion, bio-inspired sensitive thermo-responsive PNIPAM/BNNS-NH_2_ hydrogels were prepared by an in situ polymerization route. These hydrogels are hydrophilic at a temperature below LCST, and hydrophobic at a temperature above LCST. The PNIPAM/BNNS-NH_2_ hydrogels which transfer heat much faster than pure PNIPAM hydrogels can move faster under heating. The thermal sensitive hydrogels can absorb and release Rhodamine B and oil under the control of heating and cooling. These features mean the composite hydrogels are promising actuators and can find potential applications in wide areas including drug delivery and water purification.

## Supplementary Materials

The following are available online at http://www.mdpi.com/1996-1944/11/7/1069/s1, Figure S1: FT-IR spectra of (**a**) BNNS-NH_2_, (**b**) pure PNIPAM hydrogels and (**c**) PNIPAM/BNNS-NH_2_ hydrogels. Figure S2: TGA curves of BNNS-NH_2_ (**a**) and PNIPAM/BNNS-NH_2_ hydrogels with the concentration of BNNS-NH_2_ 0.06 mg/mL (**b**). Figure S3: LCST of the hydrogels (**a**) pristine PNIPAM; (**b**) PNIPAM with BNNS-NH_2_ concentration of 0.02 mg/mL; (**c**) PNIPAM with BNNS-NH_2_ concentration of 0.04 mg/mL; (**d**) the PNIPAM with BNNS-NH_2_ concentration of 0.06 mg/mL; (**e**) the PNIPAM with BNNS-NH_2_ concentration of 0.10 mg/mL; (**f**) the PNIPAM with BNNS-NH_2_ concentration of 0.20 mg/mL. (The transmittance is tested at 700 nm). Figure S4: Transmittance of dye solution after dye penetrant test (**a**) the dye solution which were put inside the hydrogels with different concentration BNNS-NH_2_ for 2 days; (**b**) the original dye solution. (The transmittance is tested at 610 nm). Figure S5: Transmittance of the dye solution after dye release test (**a**) the hydrogel without BNNS-NH_2_ in hot water; (**b**) the hydrogel with the BNNS-NH_2_ concentration of 0.02 mg/mL; (**c**) the hydrogel with the BNNS-NH_2_ concentration of 0.04 mg/mL. (The transmittance is tested at 610 nm).
