# Supplementary material for: Boron Nitride Nanosheets/PNIPAM Hydrogels with Improved Thermo-Responsive Performance
Source: Materials (Basel). 2018 Jun 24;11(7):1069. doi: 10.3390/ma11071069 (PMC6073573; doi:10.3390/ma11071069)
Supplement: Supplementary file 1 [file materials-11-01069-s001.pdf]

materials

MDPI

Supplementary

Boron Nitride Nanosheets/PNIPAM Hydrogels with Improved Thermo-Responsive Performance

Shishan Xue [1], Yuanpeng Wu [1,2*], Jiemin Wang [3], Meiling Guo [1], Dan Liu [3*] and Weiwei Lei [3*]

1 School of Material Science and Engineering, Southwest Petroleum University, Chengdu 610500, China; 201711000008@stu.swpu.edu.cn (S.X.); 201621000011@stu.swpu.edu.cn (M.G.)
2 State Key Laboratory of Oil and Gas Reservoir Geology and Exploitation, Southwest Petroleum University, Chengdu 610500, China
3 Institute for Frontier Materials, Deakin University, Locked Bag 2000, Geelong, VIC 3220, Australia; jiemin@deakin.edu.au
* Correspondence: ypwu@swpu.edu.com (Y.W.); dan.liu@deakin.edu.au (D.L.); weiwei.lei@deakin.edu.au (W.L.); Tel.: +86-028-8303-7404 (Y.W.)

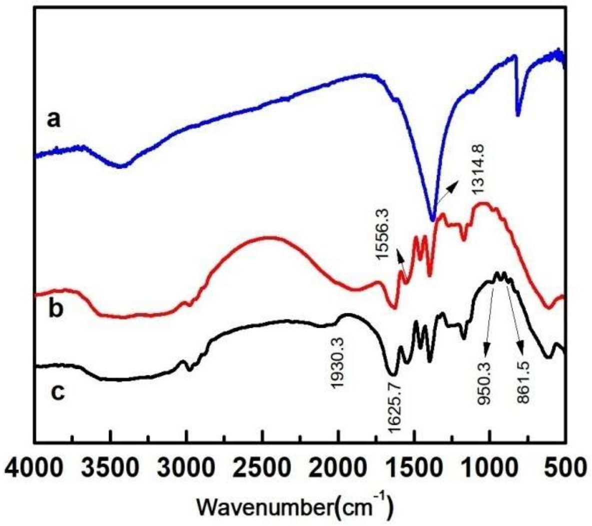

Figure S1. FT-IR spectra of (**a**) BNNS-NH_2, (**b**) pure PNIPAM hydrogels and (**c**) PNIPAM/BNNS-NH_2 hydrogels.

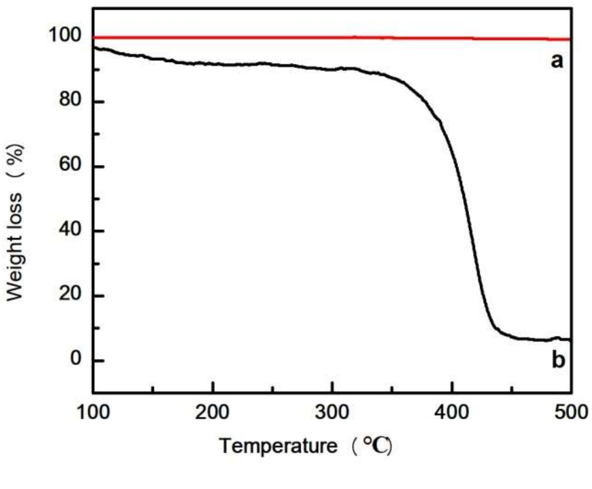

Figure S2. TGA curves of BNNS-NH_2 (**a**) and PNIPAM/BNNS-NH_2 hydrogels with the concentration of BNNS-NH_2 is 0.06 mg/mL (**b**).

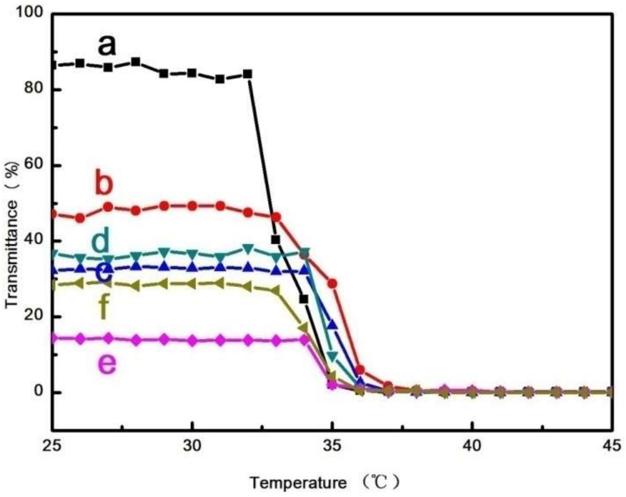

Figure S3. LCST of the hydrogels (**a**) pristine PNIPAM, (**b**) PNIPAM with BNNS-NH_2 concentration of 0.02 mg/mL, (**c**) PNIPAM with BNNS-NH_2 concentration of 0.04 mg/mL, (**d**) the PNIPAM with BNNS-NH_2 concentration of 0.06 mg/mL, (**e**) the PNIPAM with BNNS-NH_2 concentration of 0.10 mg/mL, (**f**) the PNIPAM with BNNS-NH_2 concentration of 0.20 mg/mL. (The transmittance is tested at 700 nm of UV-vis.).

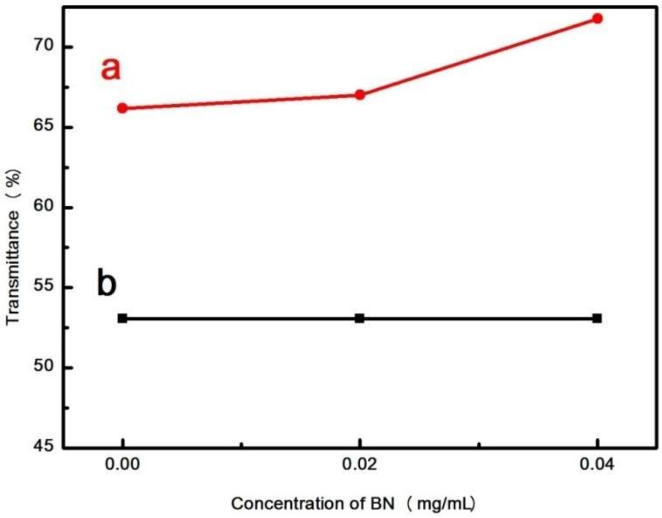

Figure S4. Transmittance of dye solution after dye penetrant test (**a**) the dye solution which were put inside the hydrogels with different concentration BNNS-NH_2 for 2 days, (**b**) the original dye solution. (The transmittance is tested at 610 nm of UV-vis).

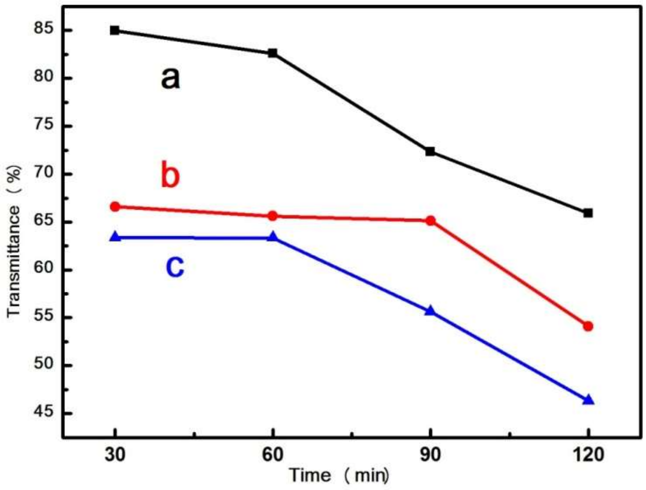

Figure S5. Transmittance of the dye solution after dye release test (**a**) the hydrogel without BNNS-NH_2 in hot water, (**b**) the hydrogel with the BNNS-NH_2 concentration of 0.02 mg/mL, (**c**) the hydrogel with the BNNS-NH_2 concentration of 0.04 mg/mL (The transmittance is tested at 610 nm of UV-vis.).

© 2018 by the authors. Submitted for possible open access publication under the terms and conditions of the Creative Commons Attribution (CC BY) license (http://creativecommons.org/licenses/by/4.0/).
